# A Practical Treatise on Midwifery, &c.

**Published:** 1845-10

**Authors:** 


I. A Practical Treatise on Midwifery, exhibiting the present Advanced State of the Science. By *F. G. Moreau*, Professor of Midwifery and the Diseases of Women and Children in the Faculty of Medicine of Paris, &c. &c. Translated from the French, by *Thomas Forrest Betton*, M.D., and edited by *Paul B. Goddard*, A.M. M.D., Lecturer on Anatomy, &c. With eighty Plates, comprising numerous Illustrations. Philadelphia, Cary and Hart, 1844. 4to, pp. 235.

II. The Principles and Practice of Obstetric Medicine and Surgery, in reference to the Process of Parturition. With one hundred and ten Illustrations on Steel and Wood. By *Francis H. Ramsbotham*, M.D., Fellow of the Royal College of Physicians, &c. Second Edition. London, Churchill, 1844.

III. De la Puberté et de l'Age Critique chez la Femme, au point de vue Physiologique, Hygienique, et Medical, et de la Ponte Periodique chez la Femme et les Mammiferes. Par M. *Raciborski*. Paris, 1844.

A Treatise on the Physiological, Hygienic, and Medical Management of Women at the Epochs of Puberty and the Cessation of Menstruation, and on the Periodical Discharge of Ova in the Human Female and in Mammifera. By M. *A. Raciborski*. Paris, 1844.

It is very gratifying to see how the obstetric department of medicine keeps pace with the increasing progress of the other branches of the science.—Its domain indeed is gradually widening; for, while midwifery, and the diseases of women and children, form as heretofore the great practical field for clinical and pathological research, the new light thrown on the physiology of conception, and the laws which govern fœtal development, at once introduce the obstetrician into the profoundest and most difficult enquiries of Physiology. An obstetric physician or practitioner, if he is just to himself and to his subject, is not a mere man-midwife. It is true that he waits upon Nature when she is performing one among the series of acts which combine in the process of reproduction. But, although as a practitioner in his relation to his patients, Parturition is isolated from the vital actions which have gone before and succeed it, yet no obstetrician, with the least pretension to be well-informed, will limit his inquiry to this single act. His pursuits begin with a knowledge of the male and female generative elements, and the mode in which, by their material contact, an ovum is fæcundated, and they end with the completion of Lactation. There is not any part or stage of this process which can be omitted. This is emphatically a subject of the present day—one of the rich bequests of the microscope. The germinal vesicle and its nucleus in the animal ovum afford a starting-point for a knowledge of the cell-theory, and the study of the way in which cells are multiplied, changed, and grouped together in the progress of development, is an interpreter of the laws of nutrition and secretion, and the unity of organisation. Now we think these great physiological points immediately concern the practical obstetrician. They ought and must be the beginning of his subject. He alone can use them. And amidst the mass of anatomical facts and observations which they include, he will find the solution and just interpretation of many of the difficulties and diseases he has to encounter. Sterility, extra-uterine gestation, abortion, monstrosities, and so on—can alone be rightly understood by a knowledge of obstetric physiology.

We have our design in making these observations. Midwifery is stigmatised as a low branch of science. It is enough for a man to practise it to be excluded from the honors of the College of Surgeons. And there is a certain superficial countenance given to this odium, in the way in which it is too frequently pursued. We know of no other branch of medicine which may unite, in an equal degree, large experience with profound ignorance. There is many a midwife in London who can number up her 1000 or 2000 cases, with no other practical result, than to have added a rash and reckless confidence to her very defective information. We encounter men too in practice, whose experience has accumulated with a large annual arithmetical progression, who yet remain wonderfully in the dark on the duties and objects of obstetric practice.

It requires little or no study to acquire a certain low taste for midwifery-practice. We have seen many a desultory loiterer at a Lying-in Charity, in full pursuit after this sort of notoriety, and we have met them in after-life, as the full-blown practitioners, all misgiving and self-distrust annihilated, and stamped and labelled with the self-adjusted title of men of great practical experience.

Now we are far from underrating Clinical Midwifery—every case is instructive—but only as it is properly followed out. We believe firmly that the antidote to nine-tenths of the meddling interfering midwifery, which is the fruitful source of uterine disease, is an experimental knowledge of Obstetric Physiology. It disciplines the mind to careful and exact observation, and in an acquaintance with the harmony, order, and vast resources of Nature in the re-production of the species, is found the best safeguard against marring and disordering her operations. We think that a practical treatise on Midwifery is not complete, unless it fairly enters into the physiology of conception, and the growth and development of the ovum.

The books which we would now notice are two Treatises on Midwifery, one by Dr. F. Ramsbotham, and the other by M. Moreau. The former is a second edition of a work, which came out in parts in 1841—the latter is an American translation of M. Moreau's work, a part of which, with the engravings, has already appeared in this country, being edited and translated by Mr. Streeter. We have added M. Raciborski's treatise, as introducing a subject of much present interest.

The second edition of Dr. Ramsbotham's work carries with it a significant proof of its value, from the fact that 2500 copies of the first edition have already been disposed of. It is not too a mere reprint of the first, for he has rendered it far more complete and useful by annexing a description of the diseases of the Puerperal and Pregnant States, with some statistical tables afforded by the practice of the Royal Maternity Charity, which he had previously published in the Medical Gazette. The treatises of both authors are embellished with numerous elaborate engravings, there being 110 illustrations in Dr. Ramsbotham's, and many more in M. Moreau's, some of which have been obviously copied into Dr. R.'s Atlas. These engravings are really well executed, and reflect great credit on the artists. They cannot fail to aid the student in understanding the subject, although, here and there, we see a little picture-making, such as the fimbriated extremity of the Fallopian tube in Plate xv. of Dr. Ramsbotham's work, which is frayed out very oddly.

The uterus performs two functions—it gives out the menstrual fluid, and protects, and incubates the ovum during gestation, casting it off during Parturition. The work of M. Raciborski has direct reference to the menstrual function—while the practical treatises necessarily consider it in its other capacity. Our readers must be well aware that the modern theory of menstruation is immediately connected with the full growth and dehiscence of a Graafian vesicle, and the escape of its contents. Dr. Power conjectured this in 1821, describing menstruation as "an effect of disappointed pregnancy;" while M. Negrier, Gendrin, Dr. Lee, and others have endeavoured to establish the same relation between menstruation and the casting off of ova, from an examination of the ovaria of women who have died during the flux. This theory has gained ground, and has been reinforced by the experiments of Bischoff and Raciborski on several species of mammals during the period of heat. The main result of these experiments is, that ova are found to be periodically cast off during the œstrum or heat, and that, too, quite independent of sexual congress. Bischoff has experimented on the bitch, ewe, pig, rabbit, and rat, in order to establish this proposition. It appears to us that he has proved it beyond all reasonable question, and also the observation which is associated with it—that the marks of the extruded ova are left in the ovaria as corpora lutea, the two corresponding in number.

The bearing which these experiments has upon menstruation in the human female, as produced by a like cause, is on the supposition that the œstrum of the subordinate mammals and the catamenial period are strictly analogous. We do not think that this is satisfactorily made out. Our authors, M. Bischoff, Pouchet, &c., have, in our estimation, adopted this analogy without securing it; and the inference with us is, that they have thereby failed to throw any light upon the cause of menstruation. There is this amount of analogy between the two—that in both there is a local flux of blood to the ovaries and uterus, and that the excitement of the ovaries in particular is visible in both. We quite subscribe to the doctrine, that the uterus merely gives out the hæmorrhage, which as a flux would be abrogated, were the ovaries deficient. This has long since been proved by the congenital absence of the ovaries in Mr. Pears' case, and the extirpation of them in Mr. Pott's case, both of them concurring with a permanent amenorrhœa; although we have sometimes been surprised at the regularity of the menstrual flux, both in its return and quantity, where both ovaries have been the subject of encysted disease, and almost every trace of their structure effaced. But the characteristic of the œstrum is the high venereal excitement which denotes the aptitude for conception. And we are told by Bischoff, Raciborski, &c., that sexual desires are especially manifested in women at the menstrual period, coincident with the supposed escape of the ovum. Nay, not only so, but that the chance of conception is greatly diminished if coitus does not take place a few days before, or within eight days after, the period—the latter being fixed as the time which the ovum requires for its transit through the tube. And M. Pouchet even regards the mid-period as affording a physical impossibility to conception.

We really do not see on what ground this notion is formed. Our own experience by no means confirms the statement, that sexual desires are rife in the human female at the monthly periods—nor that conception takes place at or near this period more than at any other time. We have known several instances where conception has occurred at a fortnight and three weeks after a flow; and M. Bischoff's limit of eight or ten days, as marking the residence and protection of the ovum within the oviduct is assuredly negatived in a wholesale way by the laws of the Jews, which forbid sexual congress until the woman has been clean for eight days. We can assure M. Bischoff, that this race is prolific enough with us, and that their progeny do not bear out the statement, that the risk of pregnancy is greatly diminished by his measure of the postponement of marital intercourse. It is to be noticed, too, that the period of heat in some animals, as the cow, mare, &c. comes on during lactation, very soon after the accomplishment of gestation. Here again is an obvious difference between the two functions. Indeed, we demur altogether to the analogy which has been instituted between the œstrum and the menstrual period. We think the experiments of M. Bischoff very valuable, as elucidating the place where the generative elements may meet and ova be fertilised; but we think any conclusion is strained and unsupported which attempts to fix similar phenomena, as he has shewn to occur at the time of heat, as the necessary concomitant of menstruation in the human female.

To us, it appears, that the most important facts on which this hypothesis of menstruation rest are derived from the post-mortem examination of the internal organs of generation during the menstrual period. Cases have been cited by Lee, Gendrin, Raciborski, Bischoff, and many others, which seem at first sight to establish a definite condition of the ovary as the uniform attendant on the menstrual flux. This condition consists in the presence of matured Graafian vesicles at the surface of the ovary ready to burst, or of the rent calices, with the aperture on the surface still visible, and leading to the emptied but vascular sac, or the various phases of reparative action around the opening. To these have been added an altered state of the lining membrane of the uterus, which is said to be raised into fungiform villi, an expansion of the Fallopian tube, and a general hyperemia of the entire sexual system. We have availed ourselves of several opportunities which have occurred to us to test the accuracy of these appearances, and the result is, that we remain sceptical about their justifying the dogma, that there is a stringent and necessary relation between the maturation of the follicles and escape of their contents, and the phenomena of menstruation. The term maturation wants defining. If it implies simply a full sized vesicle—so far near the surface as to shine through the serous and capsular envelopes of the ovary, which, on being burst, lets out an ovum with its pellucid doubled vitelline ring, surrounded by the tunic of granules and held in the fluid contained within the epithelial lining of the sac, the vascular layer of which is well covered with vessels—we do not hesitate to say that we have seen this well displayed, not only at every period of the inter-menstrual times; but even before the menses have been established. And then again, with reference to the action on the surface of the ovary, with the opening for the passage outwards of the ovum. In the first place, we do not believe this to be constant. We have undoubtedly seen the rent and beneath it an empty sac; but we have also examined the uteri of women dying during menstruation, when this has not been apparent. But, what is equally sure, we have seen the same appearances altogether independent of the catamenial time. Our own experience in the investigation of this matter has not been small, and we have found that the ovary in the general run of inspections, is rarely in a healthy state. The Graafian vesicles are altered in a variety of ways, and amongst them openings on the surface of the ovary, with subjacent vacant sacs, has been not unfrequently witnessed. Sometimes the openings are choked up with a vascular growth from below, and the appearance of a repairing rent is often given by an enlarged follicle near the surface, whose contents have been broken up and then absorbed, the falling in of the parietes, marking the factitious rent. We might greatly extend the false appearances which are seen in the ovaries, which tend materially to lessen the weight we should otherwise attach to the changes in them which have thought to be destinctive of the occurrence of menstruation.

But, secondly—it is to be noticed in Bischoff's experiments, that corpora lutea marked the site of the ova which had been cast off from the ovary. This fact is corroborated too, by Haighton's experiments on the rabbit. Where are the corpora lutea at the menstrual periods in the female? We know the office of the corpus luteum—and assuredly it is admirably designed to help forward the safe escape of the ovum from the ovary. Speaking of its function, Malpighi says "cujus ope ovulum separatur fovetur et stato tempore ejicitur." Its power of marking out and nourishing is unquestionably subordinate to the mechanism it offers for passing the ovum on to the tube. Every thing conspires to direct the impregnated ovum outwards, the vascular couch around it, the increased quantity of fluid within the follicle, possibly too the retinacula—if they really exist. Indeed, the regular uniform action of the corpus luteum in this respect, looks so much like a definite means for a necessary end, that we are loath to admit any other less regular and less apparent means for the same end. We readily admit that rents exist sometimes on the surface of the ovary, even through the capsule, without corpora lutea; but they are the results of a morbid action in the ovary, whereby its stroma is altered, and its envelopes diseased, and are widely different from openings produced by a normal process. We have never seen corpora lutea during the menstrual period—that is of a like structure and appearance as those which are seen when we *know* an ovum has been cast off after impregnation. We hope we have said enough on this subject to shew that it still needs proof, and further investigation, and that the proper subjects for the investigation and the proof are not the lower animals at the time of heat, but the human female during the menstrual periods.

Gendrin speaks of a change in the lining membrane of the uterus during menstruation, which is elevated into fungiform villi. We know this change well, and have traced its formation; but at present we forbear to say more of it, than that we have not found it a common concomitant of menstruation. The lining membrane of the womb during the flux is sometimes seen clear and smooth, when wiped with a cloth or sponge, as in the interval between the catamenia. If its tissue is pressed, a number of dark points appear, from which the menstrual fluid oozes out. And this we conceive to be the most important change in this part of the uterus. It directly concerns the alteration in its vascular structure. The fluid which comes forth is, by some, called the menstrual secretion, by others, the monthly hæmorrhage or flux, and the former sneer at the latter for adopting the views of the Ancients with reference to the discharge of blood. For our own part, we view the menstrual period as a true hæmorrhage, and that the flux itself is furnished by the open orifices of veins—not arteries. It comes from the very same veins which, when the womb is enlarged by pregnancy, are seen large and open on the inner surface of the uterus, and surrounded by its muscular fibre. We might well turn round upon the opposite party and ask them to demonstrate the glandular or other structure from whence this so-called secretion flows. We know the existence of uterine glands, but it was never contended that they furnished it.—Whence, then, does it come? On the other hand, the microscopic analysis of the menstrual fluid shews clearly that it is made up of blood-corpuscles, and the chemical analysis of it, with its peculiarity of not coagulating, exhibits nothing more than blood mixed with the mucous secretions which it meets with in its passage from the uterus to the vulva. We think, too, that the great tendency which the uterus, in its morbid states, has in causing hæmorrhage, bears a distinct relation to this natural bleeding; and we have seen that Dr. Oldham, of Guy's Hospital, has recently investigated this subject with reference to the bleedings of polypi, which he has shewn to come from the open mouths of veins in the growths themselves. Our limits will not permit us to pursue this subject further.

In the works of M. Moreau and Dr. F. Ramsbotham most of the subjects connected with Obstetric Medicine are treated of. The former is the more elaborate work, and embraces some subjects on the Art des Accouchemens which are omitted in the latter; while the supplementary Essays on the Diseases of the Pregnant and Puerperal States, in the recent edition of Dr. R.'s work, renders it the more useful and complete text-book.

We cannot forbear to notice a subject of complaint by Dr. Ramsbotham. It appears that, very soon after his book was published in England, an edition of it, with the drawings, came out in Philadelphia; and not only so, but the Editor was anonymous, and emasculated the work, by leaving out a part of Dr. R.'s annotations and the whole of his Appendix, and then, curiously enough, translated this abominable liberty into the term *revision*. It is not necessary for us to inquire whether the book would be better if shorn of a portion of the Notes and the entire Appendix, but we fully sympathise with Dr. Ramsbotham in his complaint. The Philadelphians are fond of this sort of outrage on courtesy and integrity. It is a very smart thing to appropriate another man's labour and copy his drawings, and *revise* his work for the American public. They seem to have an establishment for the purpose. No sooner does a good book come out in England, than forthwith an editor—Dr. Paul Beck Goddard, or some other—transforms it into an edition suited to the Western Hemisphere. We conclude that Dr. Paul Beck Goddard was too busy to work up Dr. Ramsbotham's book, and that some hireling, with a name not long or euphonious enough to appear, clipped it and dressed it for the Philadelphian community. This is much too bad. Mr. Curling's work on the Testis has received an American finish by Dr. Paul Beck Goddard, and one of the notices from the reviewer, designed to circulate a wretched puff of Messrs. Hart and Carey, publishers in Philadelphia, and Mr. Sherman, printer in Philadelphia, is headed with the endearing title of "Goddard's Curling." We really wish these gentlemen would leave us alone; we can readily spare their fraudulent courtesies, and we prefer to prosper or decline in our own proper person and dress, and the only thing we fear is, to be appropriated or absorbed by Dr. Paul Beck Goddard.

The Anatomy and Obstetric Properties of the Pelvis are well described in the works under consideration. Moreau's description and illustrations are very valuable, and we think Dr. Ramsbotham might advantageously have inserted the diagram which describes the circle of Carus. It is a true and accurate delineation of the course of the child through the pelvis, representing the confluence of the axes of the brim and outlet, and one which by a diagram is likely to be impressed on the minds of those learning midwifery. Moreau tells us that—

"Carus, with the intention of determining geometrically the direction of this route (the passage of the fœtus through the pelvis), advises us to trace the following curve. If we take as a centre the middle of the symphisis pubis, the very point at which the anterior extremity of the antero-posterior diameter of the pelvic excavation terminates, and for a radius the half of this diameter, *i. e.* a line 2¼ inches in length, and from this centre, with this radius, describe a circle around the symphisis pubis, it will be seen that the portion of the circle which passes over the pelvis traverses exactly the centre of the superior and the inferior straits, that in its passage across the excavation it is always over the very centre of the canal."

The section on the Functional State of the Pelvis in Moreau's work is interesting and instructive. He considers, at some length, the condition of joints of the pelvis during pregnancy, and adopts and strenuously maintains the notion of their relaxation and mobility, which he describes as an established and incontestable fact. Dr. Ramsbotham speaks of the separation as "possibly taking place in the lower animals, but certainly as not usual in the human subject." This is an undefined and wavering judgment. That it occurs in the lower animals there is not a doubt. Burdach describes at length several species in which it takes place. Our own opinion coincides with those who hold the affirmative with reference to the human female. It is one amongst the vital actions which occur during gestation. The ligaments soften and loosen, synovia is secreted in larger quantity, and hence the union of the bones is less rigid and close. All this facilitates labor—not so much by the absolute increase of the diameters of the pelvis, as by the yielding power it conveys to the joints. It is supposed that this does not take place, because, if even the symphisis pubis were separated to the extent of an inch—a very insignificant increase to the conjugate diameter would accrue from it; and again, that any augmented mobility in the joints causes severe and protracted suffering. The first is a very good reason why Sigault's operation should not be performed, but does not affect the true subject of inquiry, and there is as much difference between the normal yielding and mobility of the joints and their undue loosening and separation, as there is between the natural subsidence of the womb during the last weeks of gestation and its partial or complete prolapse—it is, in short, confounding health and disease. It is true that the former interprets the latter, of which it is nothing more than a faulty and excessive action.

Dr. Ramsbotham's work is enriched with a copy of the coloured plates of the corpus luteum from Dr. Montgomery, which must be of use to his readers.

The anatomical and physiological subjects which are involved in a consideration of the fœtal envelopes, the placenta, &c. have been partly illustrated and described by Moreau and Dr. Ramsbotham. Moreau has written a fair chapter on the development of the fœtus, which is altogether omitted by Dr. Ramsbotham. Indeed, we have no alternative than to condemn Dr. Ramsbotham's physiology as a very unsafe guide to the student, and altogether behind the present day. We regret this the more as his publisher has evidently been willing to illustrate this and every other branch of the subject, and we are solicitous that this part of obstetric science, at which William Hunter laboured so hard and effectively, should not be suffered to decline in the works and labours of his successors.

Dr. Ramsbotham, in a note, recognises Dr. R. Lee's investigations on the nerves of the uterus. We most cordially concur with him. We regard the discovery of this large system of ganglia and nervous plexuses as the most important anatomical discovery of the present day, and it is gratifying to us to pay the author of it our willing tribute of admiration for his untiring zeal and labour in displaying them. But why has not Dr. Ramsbotham, in his second edition, described them and delineated them?

The decidua is said to be "secreted—at first consisting of a tenacious fluid—and by degrees assuming the character of a perfect, organised, and tender membrane." When did Dr. Ramsbotham ever see the decidua consist of a tenacious fluid? He does not even indulge his readers with a foot-note about Weber's, Reid's, or Dr. Sharpey's views of its formation from the uterine glands, which glands are not apparently recognised by Dr. R. as constituent parts of the uterus. We do not even see a copy of Dr. Montgomery's plate of the decidua, which is sufficiently characteristic of this structure, but in its place is a bit of something which is as much like whity-brown paper as the uterine surface of the decidua.

The villi of the chorion in an early ovum are called minute-filamentous mossy vessels; and Dr. R. is tenacious about this matter, for he re-asserts it in an annotation. He says—"I look upon them as blood-vessels. They are very similar to the vascular tassels attached to the fœtal membranes which dip into the cups of the cotyledons in the gravid uterus of the cow and sheep." There is no doubt of the strict analogy between the two; but nothing can be more erroneous than to call the villi themselves bloodvessels. They are the simple sheaths which enclose the capillary loopings of the umbilical arteries, and are no more vascular than is cuticle or epithelium. A diagram copied from Wagner's Physiology would have helped the student to comprehend this distribution, which is just as demonstrable by injection under the microscope as are the villi of the intestines. The non-vascular sheath of the exo-chorion is an active cell-membrane, performing a most important function in the nutrition of the fœtus. Mr. Goodsir has lately thrown much light upon this structure.

In describing the separation between the amnion and chorion in an early ovum, Dr. R. assures us that there is a transparent watery fluid, like liq. amnii, occupying the interval. This is quite new to us. We have examined many ova, but have never met with this fluid. The interspace is in fact filled with a reticular membrane, called by Velpeau, the *corps reticulé*, which is very teasing to dissect off, and is always seen in a healthy ovum. It apparently serves as a bed to help the raising of the caudal and cephalic folds of the amnion in their growth over the embryo, and it gradually disappears with the advancing development of the ovum.

The amnion is said to have innumerable colourless vessels which secrete the liq. amnii. This we believe is mere fancy. The amnion is a cell-membrane—structureless.

Dr. R. does not appear to be more happy in his views with reference to the placenta. " It (the placenta), says Dr. R. has two faces, the one fœtal next the embryo and the other maternal in apposition to the uterus, it is covered on the fœtal face by the reflexed decidua, the chorion and the amnion." How, we ask, does the reflex decidua cover the fœtal surface of the placenta ? It cannot and does not do so ; and it is impossible to follow out the development of these parts, and not be convinced that such a notion is incorrect. The boundary of the placenta in the early ovum is the part where the decidua forms its angle of reflexion, and is raised over that portion of the exo-chorion which has not taken root within the expanded openings of the uterine glands, or, in other words, over that part which has nothing to do with the placenta.

Dr. R. tells us, that " some physiologists contend that there is a direct communication between the mother and the fœtus by means of continuous vessels. Others, that the mother's blood passes by absorption into the fœtal system. Others, again, that the mother's blood is poured into certain sinuosities or cells, existing on the maternal surface of the placenta, which are destined by Nature to receive it, and that while extravasated in these cells, the fœtal vessels deprive it of whatever is necessary for the preservation of the embryo. While another party entirely denies the existence of placental cells, and supposes that the same benefits result to the fœtus, its vessels ramifying in close approximation to those of the mother, although the mother's blood never enters the placenta, nor ever indeed leaves her system. I am myself an advocate for the last view."

We should expect that any one adopting this latter view, which to us appears quite erroneous, would at least take some pains to illustrate it. It is a question of deep interest to every obstetric physiologist; but, beyond the bald assertion of his opinion, Dr. R. does not enter into the subject. " Since the question is yet in dispute, and since its discussion would occupy much space, it would be out of place to enter upon the different arguments in a work principally directed to practical objects.",

We really think Dr. R. would have done wisely to have omitted altogether the physiological part of his book, and to have kept exclusively to the practical objects; but it is nothing short of ridiculous to shirk a question of the kind we are speaking of, because its discussion would occupy too much space, when he has not scrupled to cover nearly three pages with closely printed letter-press—amassing a heap of cumbrcus, useless, unsifted learning about the os sacrum.

Moreau has entered on the subject of the Signs of Pregnancy, which is not included in Dr. Ramsbotham's work. He divides these signs into the rational or equivocal, and the sensible, or those which are appreciated by the eye, ear, or finger. He regards the suppression of the menses as a sign worthy of great confidence, and looks upon the recurrence of the flux during the first months of pregnancy as an accident of this state.

"Admitting that we have been mistaken," says he, "and that some women do menstruate during pregnancy, we should say of these facts, as we have a right to say of those related by Deventer and Baudelocque, that they are so few in number and so extraordinary, that they can only be considered as rare exceptions to a general rule."

With Moreau we attach the utmost importance to this sign of pregnancy, when it is associated with good health, and the female is a married woman, and the flux has not been suppressed from some accidental or known cause. But, in cases of concealed or feigned pregnancy, it is obviously a sign which, depending on the testimony of the female, is quite valueless. We do not concur with our author in regarding the appearance of the flux during pregnancy as an extraordinary event. We have found it happen not unfrequently, and no enquiry of the patient, or examination of the discharge, have altered our conviction, that it is a similar discharge to the ordinary menstrual flux, recurring at like periods and in the same quantity.

Moreau's description of the areola changes is imperfect, and we think that he does not ascribe sufficient importance to them.

The following quotation is taken from his description of the general signs of pregnancy, and is worthy of attention.

"Of all the changes produced by Pregnancy, the most remarkable, in our opinion, is the modification which supervenes in the nervous system. This modification is such, that it exalts the sensibility, renders women more susceptible and more liable to the action of physical and moral agents, it changes their character; from being kind, confiding, gentle, and gay, it causes some to become hasty, irascible, jealous, peevish, and taciturn; in others it gives more activity to the intellectual faculties, and disposes them to the development of nervous affections—it stamps its seal on the diseases of pregnant or lying-in women, renders their progress more rapid, their disorders more numerous, serious, and the more dangerous, inasmuch as there is less time to prevent, understand, and relieve them. It constitutes that peculiar state called puerperal—a state which conception produces, and pregnancy develops—which the pains of child-birth increase —which continues during the child-bed state—extends to and diminishes during lactation—and ceases only when the woman has resumed her usual habits of life."

Moreau briefly sums up his estimate of the various signs of pregnancy as follows:—

"(1.) No one sign can determine with certainty a recent conception. (2.) The certainty of Pregnancy cannot be acquired by the signs called rational or equivocal. (3.) Among the sensible signs, those furnished by the eye are insufficient. (4.) The audible and tangible signs, and those derived from the fœtus can alone characterise this state. (5.) Among the last, the active motions of the fœtus may, if not lead into error, at least be mistaken for other movements produced by divers morbid states. (6.) The double pulsations of the heart, which at once denote Pregnancy, and the vitality of the fœtus, are sometimes wanting, and are never heard when the fœtus is inanimate. Lastly, the motion of ballottement is the most constant and best of all these signs, because, like the beats of the fœtal heart, it belongs only to pregnancy, exists when these pulsations are absent, and even when the fœtus is deprived of life."

There is a very full chapter in Moreau on the Classification of Labors, in which he justly gives to Solayrés the credit of establishing the relation which the presenting part maintains with the circumference of the brim, as the basis of a sound classification. Dr. Ramsbotham adopts the same classification of the vertex-presentations as M. Moreau, and they both admit that variety wherein the occipito-frontal diameter of the child's head corresponds with the conjugate diameter of the pelvis. We own our attachment to the more simple classification of M. Naegele, and we think that our authors might well have omitted the variety we have just named. To us it appears that labor is never conducted in this way, excepting under some material deviation either in the pelvis or the child's head—so decided, indeed, as to exclude such cases from natural labors. We look upon the sacro-vertebral angle as a mechanical hindrance to it, and that one of the most important functions of this promontory is to guide the head in its birth into one or other of the oblique diameters.

Dr. Ramsbotham's clinical experience on this form of presentation appears to favour the view we are advocating. "I have certainly," says he, "never been called upon to deliver by instruments when the head occupied either of the unfortunate situations now under discussion—(*i. e.* cases in which the sagittal suture runs parallel with the antero-posterior diameter of the brim)—but I have known them to obtain at the commencement of labor, and I have traced the head make a turn to one or the other side, being forced into that position by the strength of the uterine contractions in an analogous manner to the turn effected in all natural labors, when it is on the point of being expelled through the outlet."

We quite agree in this—and have met with and traced the same primary position, changed in the same way, not by the strength simply of uterine contractions, but by the guidance under the influence of these contractions of the sacro-vertebral angle, which directs the head into the oblique diameters. We believe that the head, at first, is not unfrequently in this position, but changes uniformly into the usual path. Hence we should not admit this variety into a classification of vertex-presentations. We regard, too, those presentations where the sagittal suture occupies the transverse diameters of the pelvis, as only a stage in its passage to take up the more true position in the oblique diameters. The sagittal suture may be detected indifferently running more or less obliquely at any point between the transverse and oblique diameters, varying its position in its route to the latter, but it would hardly consist with an enlarged view of the mode and mechanism of parturition, to subdivide the presentations into a like number. In cases where the head is found very high up—for instance, when the membranes have either spontaneously ruptured or have been artificially broken, having held an undue quantity of liq. amnii—we have traced the sagittal suture pretty nearly parallel with the conjugate diameter. After a pause, during which the uterus is shrinking and contracting on the child, a pain or two moves the head, and the suture is then found in the transverse diameter, and under the influence of uterine contraction it is transferred into the oblique diameter—then one or other parietal bone is found to present, denoting the altered movement of the head on its own axis, and so with a partial movement of rotation, slightly shifting the presenting part to the posterior quarter of the parietal bone, the head emerges from the outlet. All this is done surely and correctly—there is the mechanism for it—the provision against mechanical error—and that error is avoided. It is a law—a rule of nature : and as long as the pelvis and the child's head are healthy, holding their mutual and just relation, so long, we believe, there is a security against that variety of vertex-presentation, which, in the infancy of obstetric knowledge, was thought to be the invariable one, and which still holds a questioned and doubtful existence in the classification of some of the authors of the present day. Amongst the irregularities in head-presentations, Dr. R. classes those in which the anterior fontanelle is behind one or other acetabulum, or, as he expresses it, the face towards either groin. He admits the occasional three-quarters turn described by Naegele, by which the face is directed backwards ; but he thinks that this distinguished obstetric physician has overrated the frequency, as well of the presentation as of the mode of the head's passage, when it does occur.

We have already seen something of the conservative character of Dr. Ramsbotham, and we suspect that it peeps out here again. He has lost all ductility, and will not bend to any thing that looks like novelty. Thus Naegele's views are confined to one of the annotations, and he describes the diagonal positions with the face behind the groin as "tedious cases, with greater sufferings, and the time of duration more protracted than usual." We are not disposed to assent to this conclusion; for we have frequently witnessed the three-quarters turn completed, without any apparent difficulty, without augmenting the suffering or protracting the labor. One of the great practical advantages resulting from a correct estimate of Naegele's views on the mechanism of these presentations is, the reliance it induces in the resources of Nature. There is no need of interference—no need of rectifying positions. Dr. R. counsels his readers not to meddle early in the labor where the face is forwards, and behind the groin—in the hope that Nature will complete the delivery. "Presuming, however, that, after a number of tolerably strong expulsive pains, no advance takes place in the situation of the head, it will then be proper to embrace the cranium between the three first fingers and the thumb of one or other hand, and to give the face an inclination to one or other ilium, according as its original direction was to the right or left groin." The same manœuvre is to be performed when the head enters with its long diameter in the conjugate diameter of the pelvis. We firmly believe this practice to be worse than useless. There is no doubt that apparent success has frequently followed its adoption ; but the propitious result is due to natural causes, and not to the artificial means. We think it very difficult, if not impossible, to command the necessary power by the hand alone to effect this rotation where only it could be needed in the first-named position, viz. where the head is firmly wedged in the cavity of the pelvis, requiring much time to overcome the resistance. But we think it better practice to wait patiently, as in the more usual vertex-presentation, and treat both class of cases exactly in the same way. With the exception of Dr. R.'s views on the mechanism of these vertex-presentations, we think the remainder of the work on practical midwifery is in general characterised by a sound discretion, and a practical familiarity with the subjects he treats of. In speaking of that variety of breech-presentation where the face and front of the child are directed anteriorly, he says, "I believe that in no instance, if the case were left entirely to Nature, provided the child and pelvis were of common size and form, would the face be expelled under the arch of the pubes." In this we fully concur, and we only want Dr. R. to extend the same amount of implicit faith in Nature's ways and means to the conduct of a head under like circumstances. We could have well dispensed with the unsightly drawing of an operation under this form of breech case, which almost spoils and counteracts the wholesome precept we have just quoted. "When the shoulders," says Dr. R., "are about to pass, it is our duty to take care that they are offering themselves in that position most favourable for their exit; and if they be not, to turn one under the arch of the pubes, and the other into the hollow of the sacrum." And the drawing sketches the hands of the operator in an active effort to secure this turn. We think these attempts to turn about the child, under the mistaken notion of helping its exit, have been a fruitful source of bad midwifery practice in the pelvic-presentations. Moreau's plates are beautiful representations of the mechanism of breech cases, and there is happily not one where the accoucheur's hands are at work.

In the added Section on the Diseases of the Puerperal State, we find Dr. Ramsbotham separating congestion of the uterus, or, as he terms it, vascular congestion, from hysteritis. The first is a necessary forerunner and companion of the last affection, but it may fall short of it, and is readily amenable to treatment. We recognise the distinction as practical. The congestion is venous, and generally, but in our experience not always, the lochia are suppressed or scanty. There is no rigor, and but little excitement of the circulation. The uterus is tumid and tender, and contraction causes much suffering. It may follow any labor, but a lingering or instrumental labor, or where the hand has been introduced, are apt to induce it. A frequent cause of it, in our experience, is a neglected state of the bowels, and it soon yields to a free purgation or a large enema. Dr. R. leeches, and gives a brisk purgative of calomel and other cathartics, and uses emollient injections. We have rarely found it necessary to leech; for, when the bowels are cleared, and hot narcotic fomentations applied to the vulva and hypogastrium, the venous circulation is relieved, the lochia flow, and the uterus diminishes in size and sensibility.

Dr. R. regards phlegmasia dolens as one of the most interesting diseases affecting the puerperal state. He thinks the left leg is more frequently attacked than the right; and this "may probably," he says, "in some inexplicable manner, be dependent on the different distribution of the right and left spermatic vein." He thinks that those who have suffered profuse uterine hæmorrhage are frequent subjects of it. In accordance with the dissections of Dr. Robert Lee, and the author's late colleague Dr. David Davis, to whom he ascribes priority in the discovery, Dr. R. looks upon the venous system as that mainly involved in this affection; and very justly, in our minds, disclaims its near relationship with puerperal fever. It may come on in the course of puerperal fever, and be excited by a puerperal poison; but we think with our author, that there are important distinctions between them. In the treatment, Dr. R. advocates local depletion and mild aperients, for powerful cathartics are generally injurious; the limb is to be enveloped in new flannel, over which there is to be a covering of oil-silk. When the affection has lasted some days, and the pain still continues, advantage may be gained by leeching over the femoral or popliteal veins, and the use of the local vapour bath. And in the more chronic stage, frictions, with or without stimulant embrocations, and a properly adapted bandage are of essential service.

There is a useful chapter on Puerperal Mania, from which, however, we have not space to select any passages.

Dr. Ramsbotham has bestowed some pains on the chapter which treats of Puerperal Fever, and on this most perplexing and intricate disease Dr. R. has advanced somewhat novel views. It is true that he enters on the subject "with unfeigned diffidence," and "he hopes he may not be deemed presumptuous in stating," &c. &c. All this is very pretty, languishing, and lady-like, and would be very becoming in a man just past his teens, in his thesis for a degree. But when Dr. R. tells us that "his opinions are not grounded on a single series of cases, but that, having carefully watched its annual appearance in the eastern districts of London, they are deduced through the experience of a number of years, from cases occurring under many varieties of circumstances, and in all ranks of society from the most distressed pauper to persons in comfort and affluence"—we have a right to expect something which bears the mark of maturity, unclogged with trifling apologies.

Dr. R. does not like the term puerperal fever—he thinks it vague and undefined, and that it has been a snare to practitioners, and has caused fatal mistakes. It appears to him that the term has been applied to four very different diseases, which he describes under the titles of Peritonitis, Acute Tympanitis, False Peritonitis, and Typhus.

Puerperal Peritonitis appears under two forms, sporadic and contagious or epidemic, the latter of which may be communicated through the intervention of a third person—the nurse or medical attendant. Both forms are described together. The attack may be acute or insidious, and the epidemic form partakes largely of the nature of erysipelas. Among the exciting causes, the peculiar states of atmosphere are, we are sure, most properly insisted on; but Dr. R. speaks most obscurely of the immediate cause, mentioning a poisoned circulation as the opinion of others, but "with more truth and justice (he says) Locock and Ingleby regard the primary impression as made on the nervous system."

The symptoms which attend an acute case of Puerperal Peritonitis are well and faithfully described—the acute agonising tenderness, with the posture it occasions—the permanently quick pulse—hurried respiration—

short cough—peculiar countenance—the frequent suppression of both lochia and milk—the tendency to metastasis—then the period of effusion, tympanitis, delirium, sinking, and death.

"When the disorder appears as an epidemic, though the chief symptoms are still the same, many are greatly aggravated. The pulse is much more rapid than in the sporadic variety; it frequently rises at once to 140 or 150 in the minute, and is at the same time very small and easily compressed; the fever assumes more of a low type even from the commencement, and runs its course with far greater rapidity; the prostration of strength is more complete; the skin is cooler, being seldom above the natural standard; the belly becomes more early swollen and tympanitic; the breath acquires a faint and earthy odour; hiccough often occurs, from the irritability of the diaphragm, consequent on the distension of the stomach and intestines; abscesses form occasionally, either among the muscles of the extremities, or within or around the joints; and Lee, Marshall Hall, Locock, Ferguson, and Rigby, mention that the eyes, particularly the left, are sometimes attacked with a rapidly destructive inflammation; but this I have myself never observed; probably because the disease is always more severe in hospital practice." P. 586.

The morbid appearances are said to be "in different cases very generally the same." Fetid gas in the abdominal cavity, the result of rapid decomposition of the body after death—the peritoneum and omentum injected, and in patches, thickened and opaque—a varying quantity of serum gravitating towards the pelvis—patches of lymph, more or less consistent, and sometimes exclusively coating the surface of the uterus, are the evidences of peritonitis. The uterus is generally contracted—its proper structure sometimes soft and dark in colour, with pus either in its veins or its structure. The ovaries frequently are more vascular, and coated on their surface with a thick lamina of lymph. Not unfrequently they are reduced to a pultaceous mass, of a larger bulk than usual, and occasionally they are even converted into abscesses.

Sterility sometimes follows an attack of puerperal peritonitis, which may well be expected when the lesions of the ovaries, tubes, &c., are considered. In the treatment, Dr. R. is an advocate for vigorous antiphlogistic measures. Bleeding, early in the disease and largely, taken in full stream, until syncope is produced, is the first and most efficacious measure. Dr. R. thinks that this measure falls into discredit, by those who are prejudiced against it, by the blood being withdrawn either in too small quantity, or at too late a period in the disease, when in fact the stage of exhaustion has set in. After bleeding, the next object should be to purge freely. Ten or twelve grains of calomel, followed by a dose of infusion of senna or jalap, repeated every three or four hours until stools are procured; and, if the draught is rejected, an enema, or a drop of croton oil, are the means selected by Dr. R. A second bleeding may be practised if the disease, having only been suspended, should return with violence in a few hours; or, should the symptoms be mitigated, 18 or 24 leeches may be applied over the abdomen, followed by fomentations or a warm well-made linseed-meal poultice, on which may sometimes be sprinkled some spirits of turpentine. Dr. R. does not like blistering the surface of the abdomen, but he sees no objection to applying blisters to the inside of the thighs. Calomel and opium, or Dover's powder, in the proportion of three grains of the first to a quarter or half-grain of opium, or an equivalent quantity of

Dover's powder, are to be given every two or three hours, until ptyalism is produced, or the abdominal tenderness disappears.

Dr. R. does not place much reliance on emetics, and restricts their administration to cases where vomiting ushers in the disease.

"If the lochia be suppressed, or possess a bad odour, which is almost always the case, the vagina may be syringed every four or five hours with warm water, or a weak solution of the chlorates." The sparest diet ought of course to be allowed.

These active means apply only to the first stage of peritonitis. They must be changed, when the second stage or exhaustion sets in, for a stimulating and supporting plan. The disease throughout requires the most narrow watching on the part of the attendant physician.

"Every one must acknowledge that this subject is beset with great difficulties, both in description and practice; and these difficulties are increased by the fact that the various epidemics, whose histories we possess, have differed much from each other, being modified by the constitution of the atmosphere, and partaking much of the character of the then prevailing diseases. What Sydenham has designated *the constitution of the year*, has scarcely been taken into account in the treatment of these diseases; this, however, it is of the utmost importance to attend to; for we shall invariably find that, if the common fevers of the season bear depleting well, the same means will prove efficacious in arresting the puerperal diseases at the same time; while, on the other hand, if the typhoid type prevail, the lancet must be employed with a more sparing hand." P. 598.

The Acute Tympanitis of Dr. R. is nothing more than a variety of what Dr. Marshall Hall has described as "Intestinal Irritation," but he has selected the title on account of the prominent and peculiar symptom being a sudden and excessive tumefaction of the abdomen, accompanied by intense pain and depression.

The affection bears a strong resemblance to puerperal peritonitis. It begins two or three days after delivery with a severe rigor, succeeded by great heat and dryness of skin, both the rigor and heat of skin being more marked than in peritonitis. The pulse rises to 130 or 140, sometimes fluttering and tremulous, at others fuller and firmer than in peritonitis. The countenance becomes early changed, though not so anxious as in peritonitis.

"Most severe pain in the head is experienced, with intolerance of light and noise, uninterrupted wakefulness, and in many cases even delirium. Very early in the disease the abdomen swells inordinately and rapidly, becomes very tense and painful, and the transverse colon particularly can in many instances be distinctly traced: pressure aggravates the sufferings. The milk ceases to be secreted; the lochia are generally suppressed; there is great languor; an unwillingness to speak, or take nourishment; the patient lies on her back, with her legs drawn up, unsolicitous about herself, her infant, or her friends; the bowels are obstinately constipated.

"As the disease gains ground, the belly increases in size, pain, and tightness; the tongue becomes dry and brown; there is hiccough, or vomiting of offensive matter, muttering delirium, subsultus tendinum, and most of the symptoms that denote the last stage of fever; but if recovery is to be expected, the swelling and tenseness of the abdomen subside; the pain gradually goes off; the pulse becomes slower; the tongue moister; the skin cooler and softer; there is no vomiting; the intellects remain unimpaired; a desire is expressed for food; and the bowels act, together with the expulsion of a large quantity of flatus." P. 601.

" *Diagnosis.*—The symptoms of this disease are so nearly identical with those of peritonitis, that it is most difficult to draw any distinctive mark between the two. The only prominent circumstance in which any difference can be observed, perhaps, is the time when the swelling of the abdomen takes place. In peritonitis this tumefaction is the consequence of inflammatory action, and depends partly on effusion of fluid into the peritoneal cavity, and partly on inflation of the intestines; and it does not appear until the disease has existed for some time. Pain, then, is the first symptom, and the swelling occurs afterwards. But the order is reversed in tympanites; the swelling occurs here in consequence of the rapid effusion of gases, and the pain is subsequent, and produced principally by the distension of the intestines themselves, combined, perhaps, with a morbid condition of the nerves. This, although but one diagnostic sign, is the best I can point out, and will, I think, generally enable us to discriminate between the two diseases. I may add too that in peritonitis the patient generally expresses great anxiety about the result of the case; while in tympanites the nervous energy seems so deadened, that a state of perfect apathy is induced." P. 602.

The treatment is directed to relieve the over-distended state of the intestines, to increase their tone, and allay irritation. Warm carminatives and purgatives are best to be used until the bowels are emptied, and then a dose of opium. Of all internal remedies Dr. R. most depends upon the oil of turpentine in two or three drachm doses suspended in white of egg or mucilage every four hours. The diet should be nutritious, easily digestible, and food given at short intervals. Even moderate stimuli may be given. Dr. R. has seen some morbid appearances in fatal cases although comparatively few, but even these few are not mentioned.

" There is another affection of the puerperal state somewhat similar to the last in many symptoms, but differing from it in its chief feature, there being no tympanitic swelling of the abdomen. This was first described by Gooch, and is called by Ferguson *transient*, and by Locock and Rigby *false, peritonitis.*"

Dr. R. has adopted the latter title, and has described the disease as it has already been described by Ferguson, Rigby, &c. The subject itself is of prime importance; but, however perplexing individual cases may appear, the grand and broad distinction between neuralgia or irritable diseases, and those of an inflammatory nature are now well understood. No well-informed student at a hospital would think of making mere abdominal pain the ground for active depletory measures. A severe stitch in the side is not always pleuritis or hepatitis, and diffused abdominal pain, whether connected with the puerperal state or not, is not the necessary representation of inflammation.

A fever allied to typhus is that to which the term puerperal fever ought in Dr. R.'s opinion more properly be applied.

" Typhus, indeed, is very rare in the puerperal state—the most uncommon of all the affections which have been described under this denomination. It commences at the time after delivery most usual for serious disease to begin—about the second, third, or fourth day. It is perhaps ushered in by shivering, but this is by no means always the case. This is followed by the most severe pain in the head, and along the spine, accompanied with great depression of spirits; soon pain in the belly is also complained of; the abdomen swells somewhat, the countenance becomes dejected, and the eyes are very sunken. The tongue is early coated with white fur, the pulse very quick, and the skin hot and dry. There is usually suppression of milk, and sometimes of the lochia. Vomiting seldom occurs in the first stage, and the bowels are easily acted upon. When the disease is running on to a fatal termination, the tongue very soon becomes dry, brown, and raspy; the pulse is quicker and weaker; there is low delirium; the belly swells from intestinal flatus; there is vomiting of dark matter; laboured respiration; coma; and, indeed, all the symptoms of the worst kind of typhus.

"I have no doubt this disease is propagated by contagion; and it may be excited by a loaded state of the bowels, and perhaps by anxiety of mind, and other similar depressing causes.

"*Treatment.*—Except in the early stage, bleeding from the arm will be generally improper, and even then, should be restricted to plethoric patients. Our principal reliance must be placed on purgative medicines, aided by mercury and salines; but I fear the power of remedies in subduing this disease will be found but trifling. In the first instance, a few leeches may be applied to the temples or forehead, or a blister may be placed over the nape of the neck. A full dose of calomel should be exhibited, which must be followed up by purgative medicine; and after free evacuations have been procured, small quantities of calomel and opium should be given at short intervals, with saline medicines. The cathartics will generally cause the expulsion of a large quantity of dark, fætid, offensive stools, with hardened scybalæ, which have remained pent up within the bowels for a long time. I think it would be unwise to continue with the purgative plan when the intestines have been entirely unloaded, because of the depression which must accompany the drain from the membrane, but esteem mercurials and salines, or tonics, stimulants, and carminatives,—according as the disease displays features of excitement or depression,—as the most useful remedies. In the low state, bark, camphor, and ammonia, appear particularly indicated." P. 608.

Hidrosis is treated of separately, as independent of puerperal fever. It is the disease which Dr. Blundell, with strange pedantry, brought out under seven different varieties, which Dr. R. has been good enough to consolidate into one. Dr. Blundell's "ultra malignant, malignant, acute, lingering, mutable, fugacious and remittent" varieties, are all happily sweated down into a simple description of a child-bed complaint, in which copious and frequently mortal diaphoresis is the prominent, peculiar, and frightful symptom. We transcribe Dr. R's. account.

"*History and Symptoms.*—Hidrosis usually first shows itself within four or five days after delivery, and is almost invariably ushered in by more or less of rigor. Like most of the dangerous puerperal diseases, the sooner it appears after labour the more severe are the attendant symptoms, and the greater is the impendent peril. It has sometimes supervened on miscarriages of an advanced date; and, whenever I have seen it under such circumstances, it has always been preceded, and, I think I may say, excited, by a large loss of blood. Speedily after the shivering, or cold sensation, a degree of heat is experienced, which soon disappears, and gives way to an universal diaphoresis, in profuseness and intensity greatly beyond what might have been expected from the slightness of the two preceding stages. This copious sweating, far from being a relief, brings with it a feeling of the most abject depression; the pulse, which had risen rapidly, does not become less frequent as a consequence; the thirst, generally from the commencement urgent, does not abate; and no mitigation takes place in any of the symptoms. The cuticular secretion exhales a most unpleasant odour; it is very different from the pungent, acid smell, accompanying miliary fever; and yet is always equally characteristic. It most resembles the smell of newly-turned earth; and the breath also is faint and sickly. As the disease progresses the pulse continues to rise; but it often varies in a manner that is exceedingly unusual; at one period of the day being comparatively slow,—at another almost countless; and this alteration goes on day by day, until either the disease abates, or collapse and prostration supervene. Its average frequency is between 100 and 140 beats in the minute. It is seldom firm or hard, generally soft and small; but sometimes round and bounding, and yet most easily compressed. The tongue is almost always moist, and slimy; it is seldom thickly furred, nor raspy, even in the last stage; occasionally it bears throughout a perfectly healthy look; and never have I remarked it morbidly red, as it often is under intestinal irritation. The secretion of milk is soon suspended or much diminished; and the lochia either cease altogether, or, what is more common, become scanty and fœtid. The bowels act easily, and the motions are offensive, or unhealthy in colour. Diarrhœa often sets in, even early in the complaint, which it is difficult to check. The urine, notwithstanding the patient is constantly bathed in perspiration, is plentiful, and occasionally secreted in preternatural quantity. The heat of skin is not above the ordinary standard; and the sensorium is not *violently* disturbed. The state of mind, however, is often very peculiar; there is at one time present great apathy and listlessness; or perhaps no small degree of despondency and fear. At another time a quickness of manner may be remarked, or a degree of waywardness and pettish obstinacy, very foreign from the natural character; and then, again, a calm submission to every thing that is recommended to be done. In some cases that I have met with, an intense interest has been evinced in things of trifling moment; and expressions of gratitude for small services rendered have been uttered with a fervency much beyond what the occasion called for; so as to lead to the suspicion that mania would soon break out. The countenance is usually placid; though pallid, bearing its natural expression; and very different from the haggard, anxious cast of features that accompanies the malignant form of puerperal peritonitis. The patient dozes, but only for a few minutes at a time: and these snatches of sleep are not refreshing, nor followed by any abatement of the febrile paroxysm. Pressure on the general surface of the abdomen does not give pain; but if the hand be applied steadily, and with firmness, over the uterine region, some expression of suffering is called forth. As she lies quiet, however, she complains of no abdominal uneasiness: the posture generally chosen is on the back, or inclined rather to one side, with the thighs slightly flexed. Sometimes erratic pains are present; and occasionally an acute pain in the side, similar to what is felt in pleurisy. If the disease does not give way to the means resorted to, the pulse continues to increase in frequency; the abdomen, sometimes, though by no means universally, becomes tympanitic; when it does so, the evolution of gas is generally rapid, and takes place only a few hours before death. Vomiting of an offensive matter supervenes, but almost without effort, and sometimes attended with hiccough; uncontrollable diarrhœa comes on; the respiration is hurried and irregular; and the patient gradually sinks into a state of complete prostration and collapse. Low, muttering delirium mostly appears before death. In these respects the termination of hidrosis resembles that of most of the dangerous febrile diseases." P. 612.

The morbid appearances have principally been found in the uterine veins, which have been seen to contain pus, to be obstructed by coagula, and their lining membranes softened, turgid and semi-gangrenous. This, however, has not uniformly been the case, and Dr. R. relates an instance where there were no other lesions but slight abrasion and indistinct ulceration in the large intestine, although the veins were carefully examined, and their contents analysed by the microscope.

Amongst the exciting causes of Hidrosis, Dr. R. mentions loss of blood as the most frequent. " For of the many cases, " says he," which have fallen under my notice, I have not seen one where there had not been hæmorrhage during labour, and in the majority it has followed the removal of an adherent placenta by the hand introduced into the uterus."

Dr. R. does not venture to bleed in hidrosis, but "perhaps (he says) this may be attributed in those cases that I have seen, to the copious hæmorrhage that has preceded the attack." He enjoins very moderate purgation, and disapproves of mercury.

"Tonics, carminatives, and stimulants appear the most suitable remedies. Bark, especially quinine and the mineral acids, even from the onset, may be had recourse to; and the system must be sustained by any nutritious diet that the stomach can most easily assimilate. I think in most cases a liberal quantity of wine, or even brandy, may be allowed. During the progress of the case opiates will most likely be called for, either to keep down nervous excitement, to procure sleep, or to allay undue intestinal irritability; but they should not be trusted to exclusively. The good effects arising from their use will be procured, by administering them in moderate doses; and opium itself, or the salts of morphia, will be found the most beneficial of this class of remedies. Ammonia, when there is great depression, will be indicated, and it may be given in combination with the decoction of bark; or, conjoined with other stimulants, alternately with quinine and the mineral acids; or with chalk and opium, if the bowels are much relaxed." P. 617.

Our own experience of this form of disease leads us to accord with Dr. Ramsbotham in placing our reliance wholly on the supporting system. We have never thought of bleeding generally, although we have applied a few leeches low down on one or other side over the neighbourhood of the ovary, when we have detected pain there. But even this local bleeding we have sometimes wished to recal, so deep is the depression of life under these exhausting puerperal sweats. We are lavish in the use of quinine, giving two or three grains every hour, with small doses of opium in the shape of pills, which are less likely to excite the vomiting, which sometimes attends this complaint. Sponging the surface too with a solution of nitro-muriatic acid is a good local application.

We cannot, on the present occasion, enter critically into a review of Dr. R.'s four diseases which are the equivalent of the puerperal fever of other authors. We cannot say that we think he has contributed much towards elucidating the disease. We do not think that his readers will detect those varieties which are comparatively harmless from the severer sort, nor treat them any better from his description of them. For our own part we do not think we shall ever be induced to mention the term Acute Tympanites, except to reprobate it. The description of it appears to us to be any thing but the mild disease to be relieved by carminatives and aperients, which the author makes it out to be; and as to the diagnosis which Dr. R. has attempted to establish between it and Peritonitis, we think it quite unequal to the purpose. Dr. R., we believe, has missed altogether the one great clue to the comprehension of this and all the varieties of puerperal disease, namely, the infection of the blood itself. Hence it is that there is a want of reach, and clearness in his descriptions. He is like a man who has climbed a mountain until he has got into the clouds, but he cannot go higher so as to overlook them. He has lost or failed to secure a great principle, and we find the want of it in every page of these chapters. An animal poison is introduced into the system—it is so subtle that the dress or hair of a person exposed to it may transmit it to another, the only essential condition to the one receiving it, being, that she should have recently gone through labor, or the poison may have a material origin in the uterus, a putrefying placenta, or clots, &c., or pus from inflamed veins. As a poison it is carried by the circulation to all the organs and parts of the body, and its stress is felt first in one, then in another, and more in one than in another. It is sometimes so mild that it is cast out by a copious sweat or diarrhœa, by hidrosis or intestinal irritation, with or without *acute tympanites*, but in others it is so grave and injurious, that, having inflamed one or more tissues or organs, and principally that one which is nearest the seat of its absorption—it prostrates the powers of life. The treatment which is fitted to meet these varying circumstances cannot be uniform. One case will bear, and requires, active depletion, whilst another will need support. Peritonitis may exist in two cases, and yet it is quite possible for one to require a different and very modified treatment compared with the other.

We do not wish to pursue this subject further; but, since the masterly Essay of Dr. Ferguson on Puerperal Fever, we should have thought it imperative on any subsequent writer either to admit the principle he contends for, or plainly and experimentally to combat it. We don't underderstand how it can quietly be passed by.

We are much pleased to see a chapter on Scarlet Fever during the puerperal state, when this most dangerous disease becomes more dreadful. Dr. R. is an advocate for its Treatment by Ammonia, and free and watchful support. We cordially concur with him. Why did he not add a chapter on Small-pox? We have often wished to see an essay on the modifications of the exanthemata during the puerperal state, when their poison is rendered more virulent and fatal by the peculiar condition of the female.

Our limits will not permit us to examine at length the chapters on Retroversion of the Womb, Extra-uterine Gestation, or Abortion. They are in general well and clearly written, and contain much practical information.

We have engaged more critically in the review of Dr. Ramsbotham's work than in that of M. Moreau, because it is of a more recent date, more immediately concerns English students of midwifery, and has many attractions in the Atlas part of it, for popularity. Dr. Ramsbotham inherits an obstetric title, and his constant appeal to his father's opinions and experience is a graceful and most becoming tribute of respect to the distinguished author of the "Practical Observations on Midwifery."

We have freely criticised this second edition, and have marked its more prominent errors and omissions; and we hope, if the work reaches a third edition, that the former will be corrected, and the latter supplied. If the physiological portion is carefully re-written, and brought up to the knowledge of the present day, it will contribute much to extend its usefulness, and adapt it to the wants of students. But, at present, it is in this respect far behind the works of Rigby, Churchill, Campbell, and Dr. Lee. We do not hesitate, however, to recommend most of the chapters on practical midwifery as well worthy the careful study of students and practitioners, as they embody those rules and principles of treatment, which, descending to us from our predecessors, have stood the tests of time and further experience, and have been sanctioned and adopted by the teachers and writers of the present day.

